# Development of Electrolyzer Using NiCo(OH)_2_ Layered Double Hydroxide Catalyst for Efficient Water Oxidation Reaction

**DOI:** 10.3390/nano12111819

**Published:** 2022-05-26

**Authors:** Rafia Nimal, Rashida Yahya, Afzal Shah, Muhammad Abdullah Khan, Muhammad Abid Zia, Iltaf Shah

**Affiliations:** 1 Department of Chemistry, Quaid-i-Azam University, Islamabad 45320, Pakistan; rafia.numal@gmail.com (R.N.); rashidayahya34@gmail.com (R.Y.); 2 Renewable Energy Advancement Laboratory, Department of Environmental Sciences, Quaid-i-Azam University, Islamabad 45320, Pakistan; abdullah42pk@gmail.com; 3 Department of Chemistry, University of Education Lahore, Attock Campus, Attock 43600, Pakistan; abid@ue.edu.pk; 4 Department of Chemistry, College of Science, United Arab Emirates University, Al Ain P.O. Box 15551, United Arab Emirates

**Keywords:** NiCo(OH)_2_ layered double hydroxides, electrocatalysis, oxygen evolution reaction, onset potential, current density, stability

## Abstract

Over the past decade, layered double hydroxides (LDH) have been the subject of extensive investigations owing to their remarkable water splitting catalytic activity. Stability and porosity are several of the features of LDH which help them to serve as efficient oxygen evolution reaction (OER) catalysts. Based on these considerations, we synthesized NiCo(OH)_2_ LDH and probed its OER electrocatalytic performance. The synthesized catalyst was subjected to X-ray diffraction, scanning electron microscopy, and X-ray photoelectron spectroscopy for structural analysis and investigation of its surface morphology, surface composition, and oxidation states. The LDH-NiCo(OH)_2_ was anchored over the FTO surface and the fabricated electrode was found to exhibit a much lower OER onset potential of 265 mV, a much higher current density of 300 mAcm^−^^2^ and a smaller Tafel slope of 41 mVdec^−1^. Moreover, the designed catalyst was found to be stable up to 2500 repeated voltametric scans. These figures of merit regarding the structure and performance of the designed LDH are expected to provide useful insights into the fundamental understanding of the OER catalysts and their mechanisms of action, thus enabling the more rational design of cost effective and highly efficient electrocatalysts for use in water splitting.

## 1. Introduction

Energy demand has been increasing worldwide due to population growth, industrial development, and improvement in the standard of living. About 80% of this energy requirement is fulfilled by fossil fuels. However, although fossil fuels are the primary sources of energy, their reserves deplete with time. The non-sustainability of fossil fuels, and their associated environmental challenges, have prompted scientists to search out more abundant and sustainable energy sources [1,2,3]. Tidal, wind, and solar resources are renewable, clean, and eco-friendly, but their reliability is an issue due to daily and seasonal variations [4]. Hence, there is a need for a more sustainable energy source. In this regard, water splitting is a viable option, as it is renewable and abundantly available. Moreover, water splitting is a greener and more portable source, as its products can be put in storage or treated directly without creating any environmental risks [5,6]. In the 19th century, Paets van Troostwijk and Nicholson/Carlisle unveiled the method for the electrochemical splitting of water [7]. The concept of green energy relies on this discovery, and extensive efforts have been devoted to the advancement of water splitting [8,9]. However, so far, this abundant and environmentally friendly energy source has not yet been fully adopted worldwide. The reason for this is the high energy demanding anodic reaction of water electrolysis, i.e., the oxygen evolution reaction (OER). Thus, the affordability of water splitting is a challenge in the adoption of water as a greener energy source. A plethora of research teams are engaged in the search for cheaper catalysts that could trigger OER at the lowest possible potential. 

The typical electrocatalytic splitting of water is carried out in a special electrolyzer containing a cathode for water reduction reactions and an anode for water oxidation reactions. Such electrolyzers contain suitable electrolytes for the facile transportation of ions between the two electrodes [10]. Water splitting involves two half reactions: a hydrogen evolution reaction (HER) and an oxygen evolution reaction (OER) [11]. In hypothetical terms, water splitting takes place when the applied voltage reaches 1.23 V, releasing hydrogen and oxygen gases from the electrode surfaces. However, in actual practice both HER and OER demand a substantial overpotential (η) [12,13]. The OER at the anode must cross a higher energy barrier/overpotential as it has more sluggish kinetics than the HER. Therefore, water splitting efficiencies rely mostly on OER [14]. The mechanism of OER is complex and strongly dependent on the structure and properties of the electrode surface. An effective OER electrocatalyst must contain adequate adsorption sites for efficient charge transfer between the electrode and adsorbed species. Another important factor determining the efficiency of the OER catalysts is the binding strength between the electrode surface and the reaction intermediates (e.g., HO*, O*, and HOO*). Generally, OER taking place on the active sites of the electrocatalysts involves four elementary steps: first, the formation of OH* intermediate from the adsorbed water molecules; second, the decomposition of OH* to O*; third, the subsequent reaction of O* with another adsorbed H_2_O molecule to form OOH*; and finally, the formation and release of an O_2_ molecule. Theoretically, the overpotential and OER activity are governed mainly by the rate-determining step. The formation of OOH* from O* is considered as the rate determining step, as it occurs at a potential higher than 1.23 V [15,16,17,18,19,20]. Researchers are trying to design electrocatalysts which could generate an OER signal closer to 1.23 V to minimize the overpotential (which is the main reason why the use of water splitting as an energy source is so expensive). Moreover, an ideal OER electrocatalyst should be efficient, stable, abundant, and cost effective. Understanding the structure–activity relationship is crucial for designing an effective water oxidation electrocatalyst [21,22,23].

Precious metal-based materials (usually RuO_2_ and IrO_2_) have been used for a long time to attain favorable OER kinetics. However, the scarcity and high cost of noble metals hinder their large-scale use for the commercial production of renewable fuel. Therefore, for achieving sustainable OER, electrocatalysts are prepared from earth-abundant metals and their activity and stability are examined. As such, researchers are preparing electrocatalysts for water electrolysis using cheaper earth-abundant elements (Ni, Co, and Fe), rather than benchmarked expensive catalysts such as Pt [24]. Ni has emerged as a promising non-noble metal electrocatalyst for water splitting owing to its abundance, lower cost, corrosion resistance, and ability to catalyze OER at a lower overpotential. Therefore, hydroxides of Ni-based electrocatalysts have excelled amongst other options in catalyzing OER. By the incorporation of other metals, the performance of individual Ni oxides can be improved. In this regard, Co is a promising candidate for OER catalysis [25,26]. Due to a number of advantages, layered double hydroxides, which belong to the two-dimensional materials family, have gained considerable interest for use in OER since 2013. Their main advantages are their larger surface area, greater surface-to-volume ratio, significantly exposed active sites, and higher efficiency than 0D and 1D materials. Their multilayer structural adjustment can be controlled by intercalation, topological transformation, or by assembling other functional materials. Their chemical composition can be tuned by changing the ratio of cations. Their ordered porosities support the transportation of water molecules and the release of gaseous products. They have configurable active site orientation, as well as increased structural stability [27]. Hence, the current work is focused on LDH and the utilization of its unique structure for water splitting catalysis.

Multiple parameters can decide the performance of a water splitting electrocatalyst, with overpotential, current density, Tafel slope, and charge transfer resistance being the most commonly evaluated parameters for the assessment of OER performance [28,29]. Based on these considerations, we synthesized NiCo(OH)_2_ layered double hydroxide and explored its OER activity. The LDH-NiCo(OH)_2_ fabricated FTO is the first example of a highly stable, efficient, and inexpensive OER catalyst that demonstrates the low onset potential, high current density, low charge transfer resistance, long-term cyclability, and low Tafel slope desired for an OER catalyst. Hence, the results of our investigations are expected to provide valuable insights into the electrocatalytic role of LDH and improve our fundamental understanding of the relationship of structure and OER catalytic mechanism of action.

## 2. Experimental Section

### 2.1. Chemicals and Materials

Nickel(II) nitrate hexahydrate (Ni(NO_3_)_2_∙6H_2_O, purity above 97%), cobalt(II) nitrate hexahydrate (Co(NO_3_)_2_∙6H_2_O, extra pure), ammonium hydroxide of 99.99% purity, anhydrous potassium chloride of ≥99% purity, 5 μL Nafion (10% Aldrich solution), benzyl alcohol (HPLC grade), and an acetone solution of ≥99.8% purity, were all purchased from Beijing Chemicals Co., Ltd., Beijing, China. Analytical-grade ethanol was used. All these chemicals and reagents were used as purchased and solutions were made in deionized water.

### 2.2. Physical Characterization

X-ray diffraction (XRD) analysis was performed using an XRD spectrometer (D-8 Discover, Bruker, Germany) within a 10–80 (2θ degrees) range by utilizing CuKα radiation. The surface morphology was analyzed using a field-emission scanning electron microscope (JSM-7600F, JEOL, Tokio, Japan). X-ray photoelectron spectroscopy (XPS) analysis was executed by using Kratos Nova with an Al Kα energy source at 1486.6 eV. Scans were conducted at an anode voltage of 15 kV and a 10 mA current. The pass energy was set to 160 eV for survey scans and 20 eV for high-resolution scans. The binding energy was calibrated by referencing the charge to the hydrocarbon C 1s peak at 285.0 eV.

### 2.3. Synthesis of LDH-NiCo(OH)_2_

A simple co-precipitation method was used for the synthesis of layered double hydroxide. A solution was prepared by dissolving 0.64 mmol Ni(NO_3_)_2_ and 1.28 mmol Co(NO_3_)_2_ in 14 mL benzyl alcohol. The resulting solution was then stirred for 2 h at room temperature. In the next step, the solution was charged by the dropwise addition of 14 mL ammonia solution and heated to 165 °C for further two hours. The resulting precipitated solution was allowed to cool naturally and sonicated for 10 min to undo the agglomeration of particles in order to obtain a good separation. The final material was dried under nitrogen and put in a desiccator for 48 h for further drying.

### 2.4. Cleaning of the FTO Electrode

Conductive FTO glass (FTO, Omniscience, South Korea) (1 cm × 1 cm × 2 mm, 16 Ω cm^−2^) was used as a substrate for modification with the synthesized electrocatalyst. Prior to the deposition of the active material, the FTO substrate was washed properly. FTO glass was initially sonicated with acetone for 15 min and then with ethanol for another 15 min so that any organic impurity was dissolved and removed from the surface of the FTO. After thorough cleaning, the substrate was parched in a muffle furnace at a temperature of 120 °C for around 90 min for degassing. This treatment leads to a less bumpy surface and an ultimately smooth coating of active material.

### 2.5. Formation of Catalytic Ink and Working Electrode Fabrication

To carry out electrochemical studies, a working electrode was first fabricated with catalytic ink. An ink of the chosen catalyst was prepared by the accumulation of 5 mg of the synthesized NiCo(OH)_2_ in 250 μL methanol/ethanol (analytical grade) and 5 μL of Nafion binder, followed by sonication for 3–4 h. After sonication, the ink of the desired material was coated over the surface of FTO via the drop casting method. The modified FTO was then dried in oven at 70 °C for 24 h. After drying, the coated FTO (with mass loading of 0.294 mg cm^−2^) was ready for use as a working electrode.

### 2.6. Electrochemical Water Oxidation Studies

All electrochemical experiments were conducted on a Potentiostat/Galvanostat/ZRA 02529 (Interface 5000E) Gamry workstation (Warminster, PA, USA). The OER activity of the electrocatalyst was studied in 1 M KOH in a three-electrode system. The FTO coated with the desired catalyst was used as the working electrode, Ag/AgCl (3.0 M KCl) as the reference electrode, and platinum wire as a counter electrode. All the potentials in this work were measured with reference to Ag/AgCl. For conversion to the RHE scale, the following equation was used:

$$E_{\left(RHE\right)}=E_{\left(Ag/AgCl\right)}+0.197+\left(0.059\times pH\right)$$

## 3. Results and Discussion

### 3.1. Structural and Morphological Analysis 

**Figure 1:**
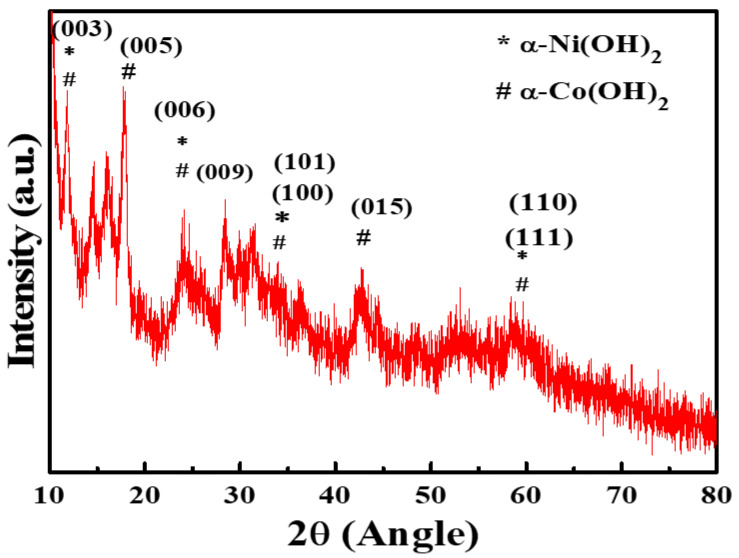
XRD pattern of NiCo(OH)_2_-LDH.

XRD was performed for the phase categorization of the synthesized material. The XRD pattern of the electrocatalyst obtained in the 2θ range of 10–80 is displayed in Figure 1.

The peaks for NiCo(OH)_2_ appear at 2θ values between 11 and 60. The detectable diffraction peaks of NiCo(OH)_2_ are in good agreement with those reported previously and can be referenced to α-Ni(OH)_2_ and α-Co(OH)_2_, matching JCPDS No: 38-0715 and JCPDS No: 01-087-0645 [30,31], respectively. The XRD pattern, which typically shows a steep rise in the low-angle section and noticeable asymmetry in the high-angle section, is suggestive of layer stacking, as observed in the case of the synthesized material. It also indicates that the synthesized material is loose and defective, with turbo static phases observed in the case of various α-type hydroxides [32,33,34]. The lesser diffraction intensity and noisy profile are indicative of poor crystallinity, as required for LDH. A loose, less crystalline structure with defective sites is generally required for good catalytic activity, as such structures provide a greater surface area and larger number of active sites for the adsorption of reactants and intermediates [35,36,37,38]. 

**Figure 2:**
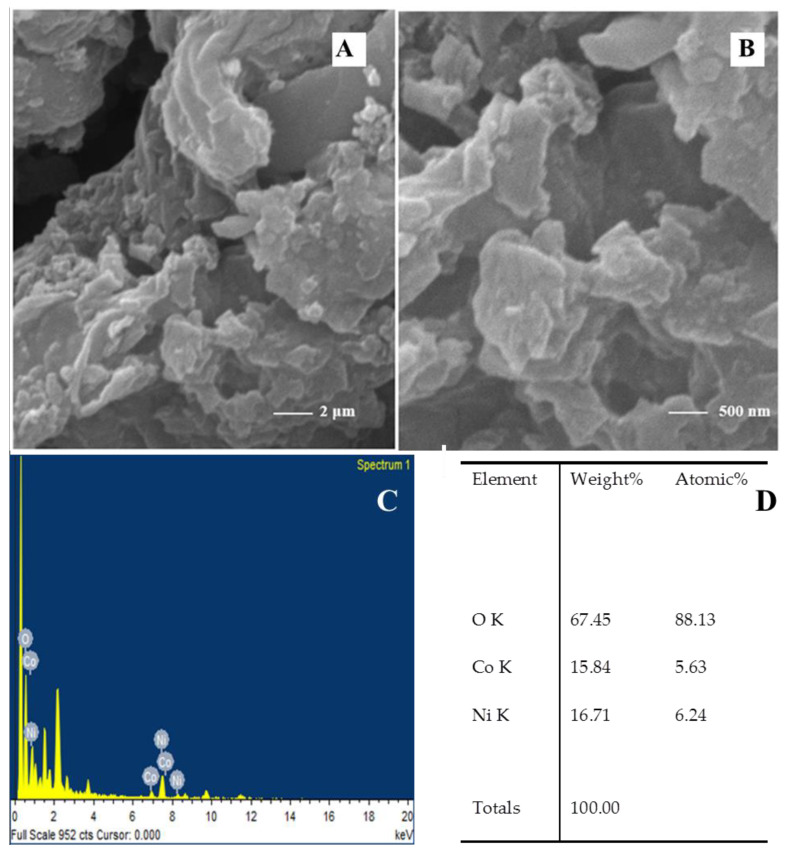
(**A,B**) SEM analysis, (**C**) EDS analysis, and (**D**) percentage of elements in NiCo(OH)_2_-LDH powdered sample.

SEM provides information about surface roughness, porosity, inter-metallic distribution, material homogeneity, and particle size. SEM was performed to analyze the surface morphology and texture of the synthesized material. The surface morphology was observed at different magnifications. Figure 2 reveals that the material has a characteristic layered structure, having pores that offer a large surface area for increased OER catalytic activity. The SEM-EDS results indicate that the Ni/Co ratio is nearing 1:1.

**Figure 3:**
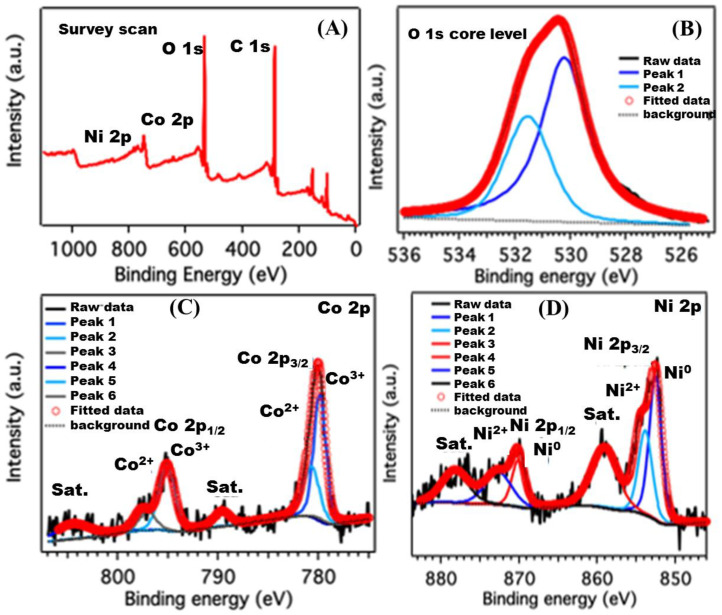
(**A**) Core-level XPS survey spectrum for NiCo(OH)_2_-LDH. The fitted and de-convoluted high-resolution XPS spectra of (**B**) oxygen are referred to as O (1s), for (**C**) cobalt are referred to as Co (2p), and for (**D**) nickel are referred to as Ni(2p).

The high-resolution Ni 2p, Co 2p, and O 1s XPS scans of the NiCo(OH)_2_ LDH sample were further investigated to determine the surface chemical states of nickel and cobalt ions, as well as the types of oxygen-containing species present (Figure 3). The O 1s signals in Figure 3B at the binding energy value of 531.2 eV and 529.5 eV can be ascribed to the oxygen atoms on the M-O-H groups of the nickel and cobalt hydroxides and the oxygen atoms on the M-O groups of cobalt oxide, respectively. The binding energy spectrum of Ni extends from 850 to 890 eV, with two clear shakeup satellites close to two spin-orbit doublets at 870.2 eV and 853 eV with a spin–energy separation of 17.2 eV, as shown in Figure 3D, and can be identified as Ni 2p_1/2_ and Ni 2p_3/2_ signals assigned to the Ni^2+^ state. Moreover, the O 1s, along with Ni 2p_3/2_ energy at 853 eV, indicates Ni-O bond formation at the surface, as reported by McIntyre and coworkers [39]. The two main peaks in Figure 3C (one at a binding energy of 781.6 eV and the other at one of 797.1 eV with two weak shake-up satellites) can be attributed to Co 2p_3/2_ and Co 2p_1/2_ orbitals. Zhang and coworkers have reported similar results for a nickel-cobalt layered double hydroxide nanoflake array, where a shoulder peak in the higher binding energy area indicates the presence of oxidized cobalt [40]. Moreover, the O 1s signal, along with Co 2p_3/2_ at 781.6 eV, suggests CoO bond formation at the surface, as reported by McIntyre and coworkers [39].

### 3.2. Electrochemical Water Oxidation Studies 

**Figure 4:**
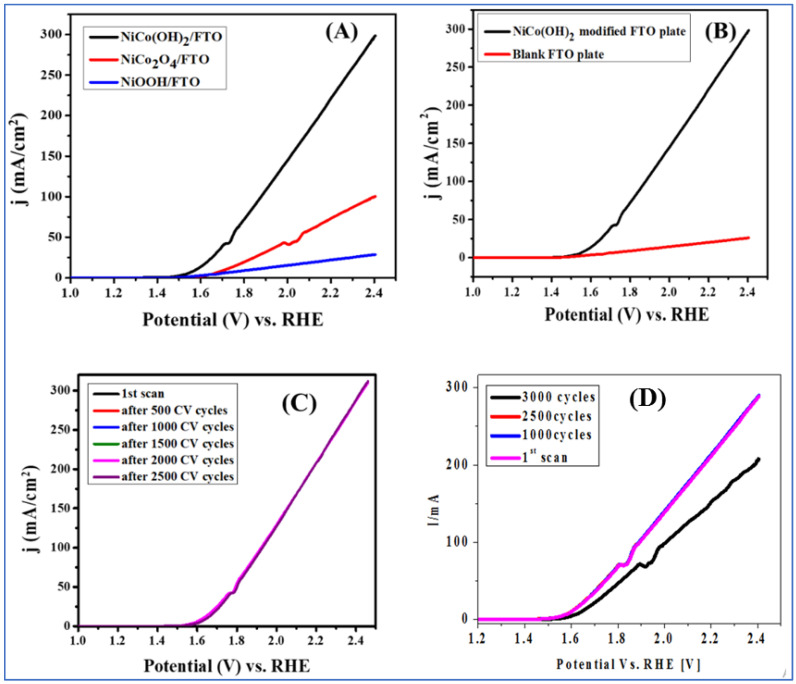
(**A**) LSV curves of NiCo(OH)_2_/FTO, NiCo_2_O_4_/FTO, and NiOOH/FTO; (**B**) LSV of NiCo(OH)_2_/FTO for OER activity in 1 M KOH solution at a scan rate of 10 mV/s; (**C**) LSVs of NiCo(OH)_2_/FTO before and up to repeated 2500 CV cycles; (**D**) LSVs showing current loss after 2500 repeated CV cycles.

Linear scan voltammetry (LSV) was used to examine the catalytic OER efficiency of NiCo(OH)_2_-LDH/FTO, NiCo_2_O_4_/FTO, and NiOOH/FTO. These three electrocatalysts were synthesized using the same co-precipitation method. By recording the LSV scans shown in Figure 4A under similar experimental conditions, it can be noted that the OER signals at NiCo(OH)_2_-LDH/FTO, NiCo_2_O_4_/FTO, and NiOOH/FTO have current density values of 300, 100 and 30 mAcm^−2^, respectively. NiCo(OH)_2_-LDH/FTO shows an onset potential of 265 mV and an over-potential of 350 mV at the current density of 10 mAcm^−2^, while NiCo_2_O_4_/FTO and NiOOH/FTO show over-potentials of 480 mV and 610 mV at the current density of 10 mAcm^−2^. The comparative study of these three electrocatalysts reveals that NiCo(OH)_2_-LDH/FTO displays the attributes of a promising OER catalyst, as is obvious due to its lower overpotential and significantly higher current density. Hence, among the three synthesized catalysts, NiCo(OH)_2_-LDH was used for further electrochemical studies. At the practical potential of 2.4 V vs. RHE, NiCo(OH)_2_/FTO registered the maximum current density of 300 mAcm^−2^. Figure 4A,B indicate that O_2_ evolution starts at a 265 mV higher potential than the theoretical value of 1230 mV. 

The electrocatalyst was tested for durability by recording LSVs after scanning consecutive CV cycles at a 50 mV/s scan rate. Before and after every 500 cycles of CV, an LSV scan was run. By plotting the LSV scans, it is seen that all 2500 curves overlap and show no loss in the current and shape of voltammograms, as demonstrated in Figure 4C. Hence, durability, which is a prime objective of the practical implementation of the catalyst, was achieved, as the synthesized catalyst qualified repeating cycling tests by retaining its efficiency even after the use of up to 2500 CV cycles. Figure 4D suggests the upper stability limit of the catalyst. 

**Figure 5:**
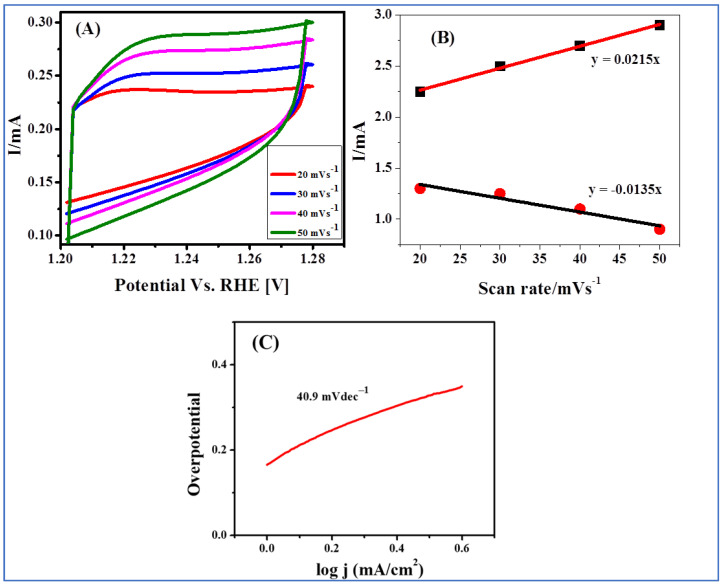
(**A**) CVs of NiCo(OH)_2_-LDH/FTO electrocatalyst performed in the non-Faradaic region at various scan rates from 10 to 50 mVs^−1^; (**B**) plot of anodic and cathodic currents against scan rate for NiCo(OH)_2_-LDH/FTO electrocatalyst; (**C**) Tafel plot based on the data (during the OER) obtained from LSV for the NiCo(OH)_2_/FTO electrocatalyst.

The electrochemically active surface area (ECSA) of NiCo(OH)_2_ (LDH) was calculated using the scan rate dependent cyclic voltammograms presented in Figure 5A. The non-Faradaic capacitive current accompanying the double-layer charging was calculated by assuming that all measured current in this range is attributable to double-layer charging. The non-Faradaic potential was set between 1.2 and 1.28 V vs. RHE. By plotting the current versus scan rate, linear plots were obtained for anodic and cathodic currents, as shown in Figure 5B. The value of double-layer capacitance (C_dl_) was determined from the average slopes. By dividing the value of C_dl_ (4 mF) by the specific capacitance C_sp_ (0.040 mFcm^−2^), the ECSA of the electrocatalyst with a value of 100 cm^2^ was obtained. This large surface area of the electrocatalyst might be one of the factors responsible for the enhanced OER electrocatalytic activity of NiCo(OH)_2_ (LDH). The catalytic roughness factor (RF), with a value of 100 cm^2^, was calculated by dividing the ECSA by the geometric area of the electrode (1 cm^2^). In general, a higher RF value is suggestive of greater catalytic activity. 

Tafel slope was calculated using the OER fitted LSV curves. For the Tafel graph, overpotential was plotted as a function of log (j), as shown in Figure 5C. Tafel slope value gives information about both the reaction kinetics and about the amount of adsorbed species generated at the surface of the catalyst. The small value (40.9 mV/dec) of the Tafel slope using data obtained at NiCo(OH)_2_/FTO indicates that the designed electrocatalyst facilitates rapid charge transport. The lower Tafel slope value demonstrates the need for a smaller overpotential, which is the key requirement for electrocatalytic oxygen evolution. Table 1 shows a comparison of the OER performance of the Ni and Co containing electrocatalysts in terms of Tafel slope, onset potential, overpotential, and maximum current density in solutions containing 1 M KOH electrolyte. 

**Figure 6:**
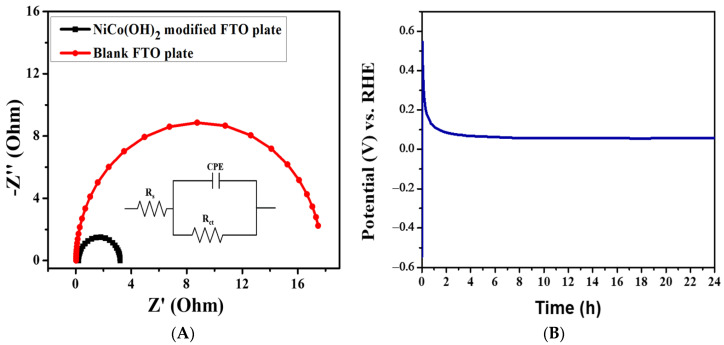
(**A**) Nyquist plot using EIS data obtained for NiCo(OH)_2_/FTO in a 1 M KOH solution. (**B**) Stability of NiCo(OH)_2_/FTO.

Charge transfer is another crucial factor for the assessment of the performance catalysts. Electrochemical impedance was performed at the bare and modified FTO under OER conditions. The EIS analysis of NiCo(OH)_2_-LDH was carried out in 1.0 M KOH solution. The respective Nyquist plot is presented in Figure 6A. The semicircular pattern reveals a property of the charge transfer mechanism. The charge transfer resistance (R_ct_), which is the resistance present between the catalytic surface and the electrolyte interface, is equal to the semicircle diameter. R_ct_ controls the OER charge transfer kinetics. In the inset of Figure 6A simplified Randles circuit (RCR) is given in which R_s_ indicates the solution resistance, R_ct_ is the charge transfer resistance and CPE is the constant phase element. A simplified Randles circuit (RCR) with resistors and capacitor was fitted to the EIS data to obtain the R_ct_, with a value of 3.7 Ω obtained for NiCo(OH)_2_/FTO. This lower value of R_ct_ is another factor responsible for the pronounced electrocatalytic activity of the synthesized catalyst.

Chronoamperometry was performed to examine the stability and durability of the NiCo(OH)_2_/FTO at a constant potential of 1.6 V for 24 h in 1 M KOH solution. The chronoamperogram shown in Figure 6B displays its stability and validates the LSV results of no current loss during the OER catalytic performance for up to 2500 consecutive CV cycles. Thus, NiCo(OH)_2_-LDH is a promising example of a highly durable and inexpensive electrocatalyst material for water oxidation.

## 4. Conclusions

An efficient, stable, and inexpensive water oxidation electrocatalyst, NiCo(OH)_2_-LDH, was synthesized via a facile co-precipitation method. The synthesized electrocatalyst was characterized through XRD, SEM, and XPS. Characterization studies revealed the formation of the catalyst in the form of layered double hydroxides. SEM results pointed to the porous and layered structure of the synthesized catalyst. XPS demonstrated the oxidation states and surface configurations of Ni and Co. Electrochemical water oxidation studies were examined for NiCo(OH)_2_/FTO through LSV, CV, EIS, and chronoamperometry. The catalytic activity of the material deposited on the FTO was measured in 1 M KOH. LSV data showed the onset potential of 265 mV, maximum current density of up to 300 A, and overpotential of 350 V at a current density of 10 mAcm^−2^ using NiCo(OH)_2_/FTO for electrocatalytic water oxidation. At the electrochemically active surface area of 100 cm^2^, the charge transfer resistance of just 3.7 Ω and Tafel slope around 41 mVdec^−1^ revealed the intrinsically favorable catalytic properties of the synthesized LDH. Chronoamperometry results and the similarity of the LSV scans of up to 2500 repeating cyclic voltammetric cycles confirmed the long-term stability of the synthesized catalyst.

## Figures and Tables

**Table 1:** Comparison of the OER performance parameters of our synthesized and reported Ni- and Co-based electrocatalysts.

Catalyst	Substrate	Current Density (mAcm^−2^)	Overpotential (η) at 10 mAcm^−2^	Onset Potential (mV)	Tafel Slope (mVdec^−1^)	Refs.
NiCo LDH@ZIF-67-V_O_/NF	NF	200	290 mV	260	58	[41]
NiCoON NSAs/NF	NF	50	247 mV	247	35	[42]
NiO/NiCo_2_O_4_	GC	70	357 mV	300	130	[43]
Ni_3_FeN	GC	150	421 mV	340	116	[44]
NiCo-S@CoFeA-TT	GCE	90	268 mV	250	62	[45]
FeNi_8_Co_2_ LDH	NF	40	210 mV	190	42	[46]
NiCo_2_O_4_/NiMn LDH	NF	80	310 mV	310	99	[47]
Fe–Co-2.3Ni–B	GC	50	274 mV	240	38	[48]
NCO−HNSs	FTO	90	340 mV	300	51	[49]
NiCo(OH)_2_-LDH	FTO	300	350 mV	265	41	This work

## Data Availability

The data presented in this study are available on request from the corresponding author.
